# Cardiometabolic Phenotype in HFpEF: Insights from Murine Models

**DOI:** 10.3390/biomedicines13030744

**Published:** 2025-03-18

**Authors:** Ekaterina Ogurtsova, Tatiana Arefieva, Anastasiia Filatova, Natalya Radyukhina, Artem Ovchinnikov

**Affiliations:** 1 Laboratory of Cell Immunology, National Medical Research Center of Cardiology Named After Academician E.I. Chazov, Academician Chazov St., 15a, 121552 Moscow, Russia; oghurtsova20@mail.ru (E.O.); tiarefieva@cardio.ru (T.A.); nradukhina@mail.ru (N.R.); 2 Faculty of Medicine, Lomonosov Moscow State University, Lomonosovsky Prospekt, 27/1, 117192 Moscow, Russia; 3 Laboratory of Myocardial Fibrosis and Heart Failure with Preserved Ejection Fraction, National Medical Research Center of Cardiology Named After Academician E.I. Chazov, Academician Chazov St., 15a, 121552 Moscow, Russia; artcardio@mail.ru; 4 Department of Clinical Functional Diagnostics, A.I. Yevdokimov Moscow State University of Medicine and Dentistry, Delegatskaya St., 20, p. 1, 127473 Moscow, Russia

**Keywords:** heart failure with preserved ejection, murine models, myocardial fibrosis, diastolic dysfunction, cardiometabolic HFpEF

## Abstract

Heart failure with preserved ejection fraction (HFpEF) remains a significant challenge in modern healthcare. It accounts for the majority of heart failure cases and their number worldwide is steadily increasing. With its high prevalence and substantial clinical impact, therapeutic strategies for HFpEF are still inadequate. This review focuses on the cardiometabolic phenotype of HFpEF which is characterised by such conditions as obesity, type 2 diabetes mellitus, and hypertension. Various murine models that mimic this phenotype are discussed. Each model’s pathophysiological aspects, namely inflammation, oxidative stress, endothelial dysfunction, changes in cardiomyocyte protein function, and myocardial metabolism alterations are examined in detail. Understanding these models can provide insight into the mechanisms underlying HFpEF and aid in the development of effective therapeutic interventions.

## 1. Introduction

Heart failure (HF) with preserved ejection fraction (HFpEF) remains a significant challenge in modern healthcare due to its complex pathophysiology and limited treatment options. It accounts for the majority of all HF cases and the number of cases worldwide is steadily increasing. This rise is attributed to factors like the gradual ageing of the population, the prevalence of a sedentary lifestyle, and the increasing incidence of cardiometabolic conditions, such as obesity, arterial hypertension, and type 2 diabetes mellitus (T2DM) [1]. HFpEF is associated with a priori high morbidity and mortality comparable to HF with reduced ejection fraction (EF) (HFrEF) [2]. According to the US Get With The Guidelines-HF registry, the 5-year mortality rate in hospitalised patients with HFpEF is as high as 75%, similar to that in patients with HFrEF [3].

HFpEF is diagnosed on the basis of HF symptoms, preserved left ventricular (LV) EF (>50%), and evidence of increased LV filling pressure/congestion with elevated brain natriuretic peptide (BNP) levels [4,5,6]. Despite the enormous clinical and social impact, therapeutic strategies for HFpEF are rather limited. This is typically a common problem owing to a gap in understanding the mechanisms underlying the development of this condition and the marked heterogeneity of its manifestations [7]. To date, regarding the drugs that have been proven to be effective in HFrEF treatment, sodium–glucose transporter 2 inhibitors and mineralocorticoid receptor antagonists are the only ones that seem to improve the prognosis of patients with HFpEF by preventing HF exacerbations [8,9,10]. In addition, the majority of pharmacological agents have no potential to improve exercise capacity and quality of life in patients with HFpEF [11]. As most of these patients are elderly with limited functional capacity due to natural age-related changes, there may be a significant problem in their treatment [8]. Moreover, the differences in drug efficacy between the two major forms of HF result from the differences in the disease initiation mechanisms. Cardiomyocyte death for any reason (ischaemic, toxic, infectious, etc.) is the key pathological process in HFrEF as it triggers the entire cascade of subsequent structural and functional myocardial changes. Regarding HFpEF, the main pathophysiological drivers are delayed relaxation and reduced LV compliance in which chronic low-grade myocardial inflammation appears to play a key role [12,13,14].

**Figure 1:**
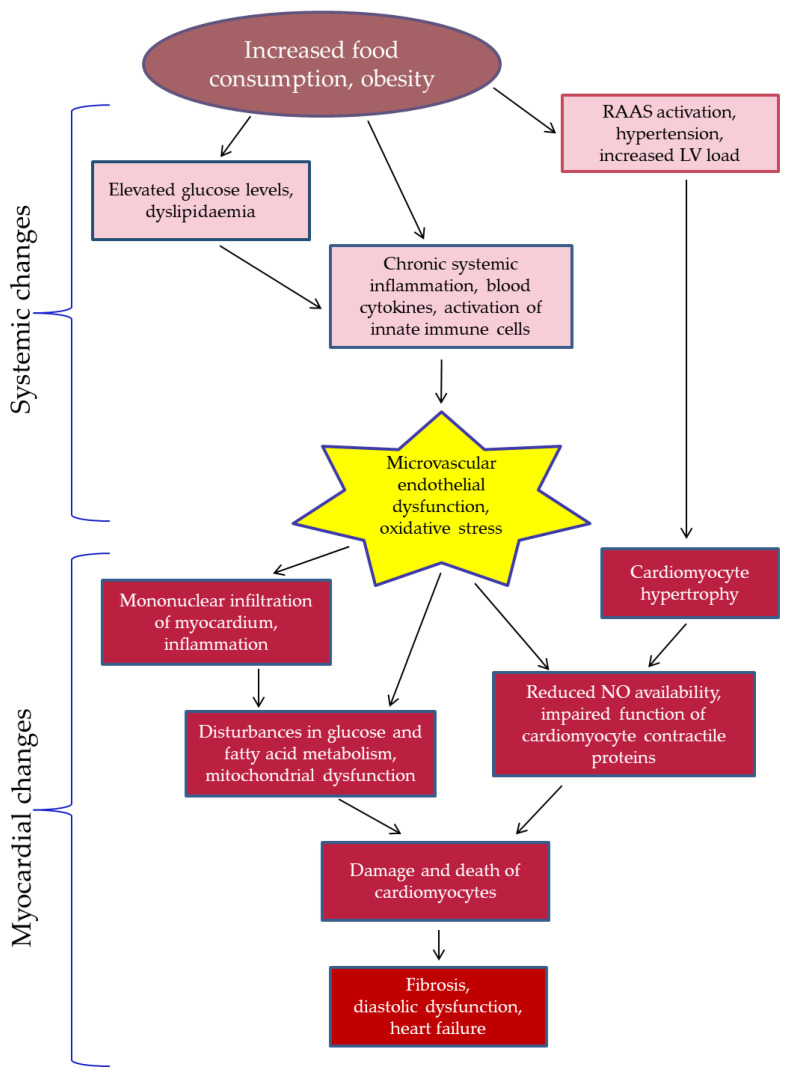
Current understanding of the pathogenesis of HFpEF. NO—nitric oxide; RAAS—renin-angiotensin-aldosterone system.

Figure 1 shows how HFpEF pathogenesis is linked to metabolic obesity, systemic inflammation, and resulting fibrosis in target organs including the myocardium. A sedentary lifestyle and overeating lead to obesity, impaired glucose and lipid metabolism, activation of the renin-angiotensin-aldosterone system, and arterial hypertension contributing to LV hypertrophy. Adipose tissue releases inflammatory factors, including cytokines and adipokines, causing chronic systemic inflammation indicated by elevated blood concentrations of cytokines and activated immune cells [2]. Oxidative stress and endothelial dysfunction are crucial to metabolic disorders and cardiovascular diseases. Excess reactive oxygen species (ROS) from immune cells activate inflammatory pathways, reducing nitric oxide (NO) availability and impairing endothelial function. Factors like hypertension, hyperglycaemia, dyslipidaemia, and smoking worsen endothelial function which can further exacerbate oxidative stress via inflammation and additional ROS production, creating a vicious cycle that contributes to the progression of cardiovascular disease. The consequences of endothelial dysfunction encompass vasoconstriction due to impaired NO production, increased permeability to inflammatory cells and lipids, and an elevated risk of thrombosis [15]. Oxidative stress-related impairment of NO production by the endothelium results in the dysfunction of the NO-cGMP-PKG signalling pathway in cardiomyocytes. PKG phosphorylates various target proteins, leading to multiple effects: inhibiting L-type calcium channels, enhancing intracellular diastolic calcium reuptake via phospholamban phosphorylation, suppressing prolonged signalling by inhibiting G-protein-coupled receptors and the transient receptor potential canonical channels, inhibiting ischaemia-reperfusion injury through ATP-sensitive potassium channel phosphorylation, and stimulating LV relaxation by phosphorylation of troponin I and titin [16].

Taken together, these mechanisms lead to cardiomyocyte death and fibrosis, which result in impaired myocardial contractility.

Clinical proof of HFpEF’ inflammatory nature

Several clinical trials have confirmed the role of inflammation in the development and progression of HFpEF. To describe the main manifestations of HFpEF, their key characteristics are provided below.

High blood levels of inflammatory cytokines. Patients with HFpEF are well known to have significantly higher blood levels of pro-inflammatory and pro-fibrotic markers, lower levels of markers of myocardial damage (high-sensitivity troponin T) and LV wall stress (N-terminal fragment of brain natriuretic hormone precursor, NT-proBNP) compared with patients with HFrEF [17,18,19,20,21]. In addition, elevated blood levels of interleukin(IL)-2 and C-reactive protein are associated with new-onset HFpEF [22,23]. Thus, the intensity of systemic inflammation significantly influences the course and prognosis of HFpEF [22,23,24,25].

Activation of endothelial cells. In patients with HFpEF, high levels of endothelial-derived molecules (intercellular adhesion molecule-1, vascular cell adhesion molecule-1, E-selectin) identified in peripheral blood and homogenates of myocardial specimens indicate endothelial cell activation [26,27,28]. Since circulating pro-inflammatory cytokines primarily affect microvascular endothelial cells, the activity of nicotinamide adenine dinucleotide phosphate oxidase (NOX), the enzyme that leads to the accumulation of ROS in cells, is increased [28]. This leads to uncoupling of endothelial nitric oxide synthase (eNOS) and decreased nitric oxide (NO) bioavailability. The above findings are supported by the low myocardial nitrite/nitrate concentrations in HFpEF patients [28]. Moreover, the activation of endothelial cells in HFpEF is associated with structural microvascular changes, such as rarefaction and basal membrane thickening of the microvasculature [29,30] which reduce coronary reserve and cause relative myocardial ischaemia.

Activation of circulating monocytes and monocyte infiltration of myocardium. Patients with HFpEF are characterised by an increased content of pro-inflammatory monocytes in the peripheral blood, translating to LV dysfunction [31]. Our recent findings show that myocardial remodelling in HFpEF patients is associated with increased numbers of circulating intermediate monocytes and a decrease in the ratio of regulatory T cells/activated T helper cells [32]. According to other research, stimulation with tumour necrosis factor (TNF) or lipopolysaccharide results in the release of large amounts of pro-inflammatory cytokines by neutrophils from HFpEF patients compared to neutrophils isolated from healthy subjects [26]. In addition, a significant accumulation of activated monocytes/macrophages migrated from the bloodstream is consistently found in myocardial biopsy specimens from patients with HFpEF [27,33,34]. Chemoattractants (mainly monocyte chemotactic protein-1 (MCP-1)) are also required for the migration of monocytes from the bloodstream into the subendothelial space of the myocardium, the initial key step in the inflammatory process. One must note that peripheral chemokine concentration (CCL2/MCP-1, CXCL10/interferon-γ-induced protein 10, CCL17, CCL18) is increased in HFpEF [31]. Notably, the numbers of classic/pro-inflammatory monocytes and alternative/anti-inflammatory circulating monocytes are increased in HFpEF patients. The culture of healthy donor monocytes with HFpEF patient-derived sera leads to M2-macrophage differentiation (as evidenced by the CD206 and IL-10 expression) [31]. According to the data of Bajpai G. et al., CCR2+ macrophage abundance is associated with LV remodelling [35]. In patients with HFpEF, transforming growth factor (TGF)-β-producing macrophages are detected in the myocardium. Moreover, the addition of TGF-β to the culture of fibroblasts obtained from patients with HFpEF is associated with their transformation into myofibroblasts. A positive correlation between cardiac collagen as well as the number of inflammatory cells, and diastolic dysfunction suggests a direct influence of inflammation on fibrosis triggering diastolic dysfunction [27,34].

HFpEF is a heterogeneous disease, mainly with cardiometabolic phenotype

Drug interventions in HFpEF are often ineffective due to the wide variability among patient phenotypes. These phenotypes vary greatly in clinical and haemodynamic features, development mechanisms, prognosis, and response to treatments [36,37,38,39]. Such heterogeneity is best assessed using sophisticated approaches (e.g., machine learning techniques). Since 2014, several research groups have applied machine learning to identify multiple clusters/phenotypes of HFpEF. To date, around two dozen phenotypes have been proposed. From study to study, the phenotypes vary, but in most cases, they are reduced to a small number of fairly well-defined phenotypes: cardiometabolic, left atrial myopathy phenotype, natriuretic peptide deficiency phenotype, and pulmonary hypertension/right ventricular dysfunction phenotype [40].

One of the most common HFpEF phenotypes mentioned above is cardiometabolic (or inflammatory-metabolic) [7]. The main risk factors predisposing to the development of HFpEF are pro-inflammatory conditions such as obesity, T2DM, arterial hypertension, chronic kidney disease, hypodynamia, and ageing [37,41,42,43]. They often combine (especially obesity and T2DM) exacerbating the role of each other [36,38]. As obesity continues to rise, it increases the amount of adipose tissue secreting pro-inflammatory adipokines, which provokes the development of insulin resistance and oxidation processes caused by free radicals, the production of pro-inflammatory cytokines, dysfunctional and reduced microvascular density, increased leukocyte adhesion, macrophage activation, and myocardial fibrosis [2,14,44,45,46]. Insulin resistance and T2DM are rather common in HFpEF indicating a worse prognosis [47,48]. Irrespectively, T2DM leads to impaired diastolic and systolic LV function with the development of the so-called diabetic cardiomyopathy regardless of other diseases such as arterial hypertension and ischaemic heart disease [49]. Hypertension is also to some extent a pro-inflammatory state. Increased blood levels of the pro-inflammatory cytokines IL-1β, TNF, and interferon-γ have been proven to confirm the risk of arterial hypertension [50]. An increase in the number of T helper 17 cells and in the concentration of cytokines produced by these cells, IL-17 and IL-23, has been reported in the blood of patients with arterial hypertension [51]. One of the key mechanisms implicated in blood pressure increase is the hyper-activation of the renin-angiotensin-aldosterone system. Moreover, angiotensin II, through its type 1 receptors, promotes inflammation in the myocardium by triggering intracellular signalling pathways leading to NF-κB activation and subsequent production of pro-inflammatory cytokines, synthesis of adhesion molecules, and oxidative stress [52,53].

In patients with the cardiometabolic phenotype, the inflammatory-metabolic milieu of obesity and T2DM comorbidities is thought to be a major contributor to the pathophysiology of HFpEF [42]. Interestingly, this phenotype is more common in women, who are more prone to systemic inflammatory diseases than men [5,36,38,54]. Packer M. et al. considered obesity and T2DM to have a greater impact on the woman’s heart than the man’s heart, HFpEF being clinically more severe. However, the prognosis for women is more favourable than for men [55]. The cardiometabolic phenotype is characterised by pronounced LV hypertrophy and diastolic dysfunction, and a high incidence of adverse outcomes [38,39,40,55,56,57]. As the vast majority of HFpEF patients are overweight/obese, this phenotype is to some extent universal [58]. Therapeutic interventions for this phenotype may be useful in the vast majority of patients with HFpEF. In other words, a “one-size-fits-all” approach is being implemented, as to some extent is suggested by the success of sodium–glucose transporter 2 inhibitors in HFpEF [9,58].

Therapies targeting inflammation in HF

The majority of clinical trials assessing the impact of anti-inflammatory therapy in HF have primarily focused on patients with HFrEF and have not demonstrated definitive beneficial outcomes. This is particularly evident in trials involving TNF inhibitors, corticosteroids, xanthine oxidase inhibitors, immunoglobulin G3 (IgG3) immunoadsorption, and methotrexate. To date, canakinumab, a monoclonal antibody targeting IL-1, is the only treatment that has shown a reduction in HF hospitalisation risk among patients with previous myocardial infarction in the CANTOS trial; however, it has not been evaluated in patients with HFpEF [59]. Results from the phase 2 D-HART-2 study in HFpEF patients indicated that IL-1 blockade using anakinra (a recombinant human IL-1 receptor antagonist) did not enhance peak oxygen consumption but was linked to reductions in blood BNP and C-reactive protein levels [60]. The SATELLITE study, which concluded early after randomising 41 patients, established the safety of the myeloperoxidase inhibitor AZD4831 in patients with HF and LV EF of ≥40%; it also demonstrated a more than 50% decrease in myeloperoxidase activity from baseline [61]. Our preliminary data suggest that the use of statins in HFpEF was associated with reductions in systemic inflammatory markers (C-reactive protein, MCP-1) and improvements in 6-min walk distance and E/e′ ratio. This supports the previously recognised anti-inflammatory properties of statins and endorses their use in HFpEF management. Currently, the phase 2 clinical trial COLHEART-PRESERVED (NCT06081049) is investigating the efficacy of colchicine in HFpEF. Notably, the ongoing large randomised phase 3 clinical trial, HERMES (NCT05636176), aims to determine the effect of the IL-6 inhibitor ziltirakimab on prognosis in HFpEF.

In summary, despite the established role of inflammation in HFpEF pathophysiology, there is a lack of studies evaluating anti-inflammatory therapies specifically for this condition.

## 2. HFpEF Animal Models

At present, the availability of suitable animal models of HFpEF throws light on understanding the pathophysiological mechanisms of HFpEF and testing new pharmacological agents. Moreover, pharmacological or surgical approaches, genetic modifications as well as their possible combinations are commonly used in the development of preclinical models of HF [62]. Both small (rodents, guinea pigs) and medium or large (dogs, cats, pigs, monkeys) laboratory animals may be the subject of such manipulation. Yet, a large number of preclinical models that reproduce aspects of HFpEF are also available to researchers [62,63,64]. For a number of reasons, mice are most commonly used to mimic HFpEF. Firstly, from a practical point of view, mice seem to be optimal animals for carrying out different research: they are cheap to maintain, have a short breeding cycle, develop pathological changes rapidly, and provide easy access to genetic manipulations. Secondly, the mouse genome proves to have sufficient homology with the human genome. However, one of the major challenges of animal models is the fact that none of them are able to fully reproduce the pathophysiological diversity of human HFpEF. Additionally, the heterogeneity of HFpEF manifestations represents another difficulty in creating animal models. Thus, no animal model is suitable for all patients with HFpEF, but for a certain group of patients. In addition, even numerous preclinical models of concentric LV hypertrophy have been a hallmark of HFpEF as they are unable to reproduce the complex myocardial remodelling observed in humans with HFpEF. Usually, the development of animal models of HFpEF has focused on recapitulating the pathology of a single organ (typically the heart) making it difficult to mimic the numerous comorbidities associated with HFpEF [65]. However, the last decade has witnessed the development of innovative multi-component animal models of HFpEF that are able to reproduce the multiple comorbidities and multi-organ involvement typical of HFpEF. Such multi-component models include two-hit models (mimicking two cardiometabolic stresses, that is, arterial hypertension, T2DM or obesity [66,67,68,69,70]). However, three-hit models are also developed including age as an extra risk factor for cardiometabolic stresses [71,72,73,74,75].

As the cardiometabolic phenotype is to some extent a universal phenotype in HFpEF [7], rodent models that recapitulate cardiometabolic stress promise an extra opportunity for the study of HFpEF. In this review, the following murine models that are more or less consistent with the cardiometabolic phenotype of HFpEF are as follows:

(1) Obesity-related HFpEF (leptin-deficient ob/ob and leptin receptor-deficient db/db mutant strains; induced by high-fat diet (HFD));
(2) HFpEF induced by arterial hypertension (chronic subjection to deoxycorticosterone acetate (DOCA), DOCA + angiotensin II infusion, transverse aortic constriction (TAC), chronic subjection to angiotensin II, DOCA infusion + TAC);
(3) Ageing HFpEF (senescence-accelerated mouse strains).

For each of these models, the individual pathophysiological aspects of the cardiometabolic phenotype are characterised below:

(1) Inflammation (production of pro-inflammatory cytokines and activation of inflammatory cells);
(2) Oxidative stress (dysfunction of the antioxidant systems and overproduction of ROS, mitochondrial dysfunction);
(3) Dysfunction of the NO/cyclic guanosine monophosphate (cGMP)/protein kinase G (PKG) pathway (decreased PKG and eNOS activity, activation of inducible NOS (iNOS), decreased cGMP and NO production);
(4) Changes in the level of phosphorylation in cardiomyocyte proteins involved in contraction and regulation of intracellular calcium waves (titin, myosin heavy chain (MHC), sarco-endoplasmic reticulum Ca^2+^-ATPase (SERCA), phospholamban);
(5) Changes in myocardial metabolism (suppression of glycolysis, increased fatty acid uptake and oxidation, impaired processes of oxidative phosphorylation and ATP synthesis).

### 2.1. Obesity-Related Models of HFpEF

Given the high prevalence of obesity in patients with HFpEF, its increasing burden contributes to cardiovascular dysfunction.

#### 2.1.1. Diabetic db/db and Obese ob/ob Mice

**Figure 2:**
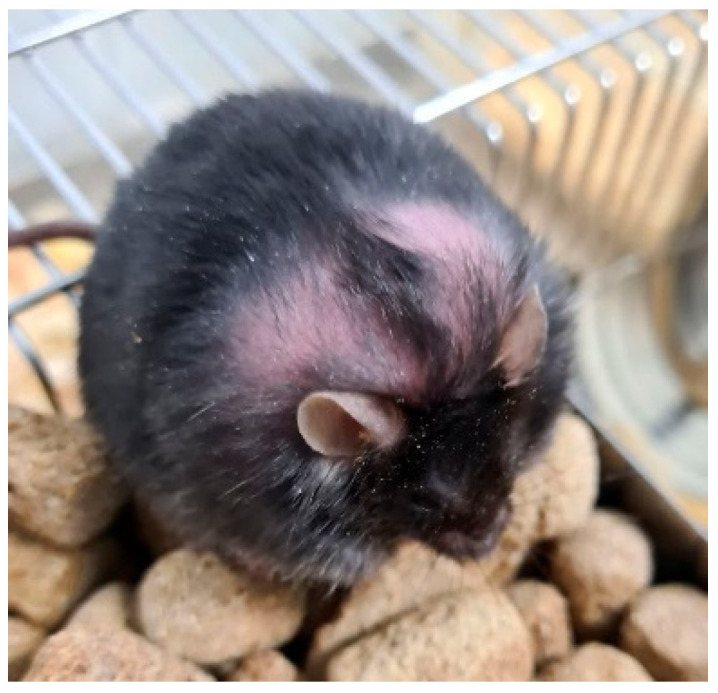
Twelve-month-old female db/db mouse (weight 63 g). The mouse was sourced from the SPF vivarium of the Institute of Cytology and Genetics, Siberian Branch of the Russian Academy of Sciences.

db/db [BKS-Lepr/db/db/JOrlRj, BKS.Cg-Dock7m+/+Leprdb/J] (Figure 2) and ob/ob [B6.V-Lepob/ob/JRj, B6.Cg-Lepob/J] strains are originally utilised as models of T2DM. Both strains are based on impaired signalling of leptin which represents a small adipokine protein with hormonal properties. As it is synthesised by adipocytes, its level in blood correlates with adipose tissue mass (it is higher in women because they have more excess adipose tissue). The leptin receptor is expressed in various tissues (heart, muscle, lung, small intestine, liver, adipose tissue, central nervous system) and is able to cross the blood-brain barrier and interact with receptors in the hypothalamus to induce a feeling of satiety, thereby regulating appetite. It is worth noting that mutations in the leptin gene or its receptor related to loss of function lead to uncontrollable hunger [76]. There is also a mutation in the db/db mice gene encoding the long isoform of the leptin receptor [77]. In this mouse strain, impaired leptin signalling in the hypothalamus leads to hyperphagia and obesity accompanied by elevated levels of insulin and leptin, hyperglycaemia, and the development of insulin resistance [78,79,80,81]. Experimentally, by 6–8 weeks of age, db/db mice suffer from significant hyperinsulinemia, obesity, and hyperglycaemia which worsen thereafter [78,79,82,83,84,85,86,87,88,89,90,91,92,93].

Another murine strain, ob/ob, has genetic leptin deficiency which can be supplemented externally [94]. Thus, they suffer from obesity, hyperphagia, hyperglycaemia, insulin resistance, and hyperinsulinaemia from an early age [81,95,96,97,98,99,100,101,102]. Impaired glucose tolerance in ob/ob mice occurs at the same age as in db/db mice, at about 6-8 weeks [80].

Both db/db and ob/ob mice demonstrate numerous signs of disturbed lipid metabolism, both models being characterised by hypercholesterolaemia and an increase in plasma fatty acids and triglycerides [78,80,82,84,90,91,98,102,103]. However, these changes are more pronounced in the db/db strain. Moreover, only db/db mice show an increase in plasma ceramide levels [78,91]. Other data report that ob/ob mice have normal blood triglyceride levels, but liver steatosis can be elevated [100,101]. Db/db mice also demonstrate increased very-low-density lipoprotein and decreased high-density lipoprotein plasma levels [103]. In addition, db/db mice have a reduced activity of hepatic triglyceride lipase which makes them similar to people with T2DM [82]. In db/db mice, a decrease in lipoprotein lipase mRNA content and activity is observed in all tissues [82].

However, there is conflicting evidence regarding metabolic disorders in the myocardium of these murine strains. For example, some authors have described high levels of triglycerides and cholesterol in the myocardium of db/db and ob/ob mice [81], particularly in ob/ob females [101], while no findings have been confirmed by Barouch L.A. et al. [95]. In the myocardium of ob/ob female mice, increased mRNA content of lipoprotein lipase and other proteins involved in lipolysis is found, as well as an increased mRNA level of the insulin-sensitive glucose transporter GLUT-4 and the microsomal triacylglycerol-transport protein which has a protective function in the presence of excess lipids [101]. Increased expression of sodium-glucose cotransporter 1 is also observed in the myocardium of ob/ob mice of both sexes [102].

A decrease in the level of aerobic glucose oxidation and glycolysis in the myocardium is observed in both strains [80], while db/db mice also confirm an increase in the levels of acetyl coenzyme A and succinyl coenzyme A, crucial metabolites in the tricarboxylic acid cycle [78]. All these factors point to a substrate shift in the energy supply of cardiomyocytes, switching from glucose oxidation to beta-oxidation of fatty acids. In addition, there is extra evidence of a disruption in the intracellular insulin signalling pathway in both strains. For example, db/db mice demonstrate an impaired response to insulin stimulation [78] and increased serine residue (307) phosphorylation on insulin receptor substrate-1 (IRS-1) (a protein that plays a pivotal role in insulin signalling) in cardiomyocytes, with subsequent inactivation of the insulin receptor [80]. In addition, these mice have reduced activity of SIRT [silent mating type information regulation 2 homolog 1], which is responsible for the ‘beneficial’ tyrosine phosphorylation of IRS-1 required for normal insulin receptor function [91]. An increase in the serine-phosphorylated IRS-1 has also been found in ob/ob mice [96]. In general, impaired cardiac metabolism precedes the onset of hyperglycaemia and the development of insulin resistance in both db/db and ob/ob mice [80].

It is also worth mentioning that leptin is important for the maintenance of normal cardiac structure and function as it may have both direct and indirect (via neurohormonal regulation) antihypertrophic effects. Hence, leptin deficiency leads to LV hypertrophy [95,97]. ob/ob mice exhibit LV hypertrophy at 6 months of age, regardless of their weight [95]. Although weight loss by dietary restriction is associated with a reduction in LV hypertrophy, a much greater reduction in LV hypertrophy may be achieved by leptin administration [95]. In ob/ob mice, the cardiac response to beta-adrenergic stimulation was also reduced; administration of leptin partially restored the beta-adrenergic inotropic response [98].

In addition to HFpEF, some comorbidities consistent with those observed in humans are developed in these murine strains, the common comorbidities being the cardiometabolic phenotype of HFpEF which usually occurs in combination with obesity, T2DM and arterial hypertension. For example, in db/db mice, the development of arterial hypertension with age is associated with obesity and T2DM [83,85,88,92]. There is an increase in blood pressure starting at 11 weeks of age, and pronounced arterial hypertension is already present by 14 weeks of age as well. Developing arterial hypertension is associated with increased renin-angiotensin system activity while the baroreflex is not impaired. It is also worth noting that in db/db mice, renin-angiotensin-aldosterone system blockers do not improve metabolism and insulin sensitivity, i.e., they have no effect on hyperglycaemia [83]. Unlike db/db mice, blood pressure is not increased with age and may be even decreased in ob/ob mice [95,101]. Such differences may be related to the fact that the authors of ob/ob mice studies have measured blood pressure in rather young mice (not older than 10–11 weeks), whereas the authors performing db/db mice studies measured blood pressure in aged mice (10 to 20 weeks). Perhaps, ob/ob mice develop arterial hypertension at a more “mature” age, especially taking into account that all the changes in these mice occurred later and were less pronounced than in db/db mice.

One more critical component of the pathogenesis of HFpEF is chronic low-grade inflammation [12] related to both db/db and ob/ob strains. It is also worth noting that the inflammation can be both systemic and myocardial. For example, the blood levels of the pro-inflammatory cytokines TNF and IL-6 are increased in db/db mice [103] and C-reactive protein—in ob/ob mice [102]. In the myocardium of ob/ob mice, RT-qPCR reveals increased mRNA of IL-1β, IL-6, TNF, NLRP3 and caspase-1 [102], and increased expression of the *TNF* and *MCP-1* genes in db/db mice [87]. According to other data, TNF, MCP-1, IL-1β and IL-6 mRNA content is increased in the adipose tissue but not in the myocardium of db/db mice [78]. However, no findings of any infiltration of the myocardium by macrophages are confirmed by the same authors [78]. In db/db mice, the enhanced NF-κB activity (assessed by the binding activity of free NF-κB p65 in nuclear extracts) and NF-κB p50 expression (determined by RT–qPCR and western blot) in LV tissue is detected. This was associated with increased ROS, superoxide, and peroxynitrite production by mitochondria. The addition of an NF- κB antagonist reduced the production of pro-inflammatory cytokines, protected the heart from oxidative stress, restored mitochondrial integrity, and reduced myocardial dysfunction [103].

**Figure 3:**
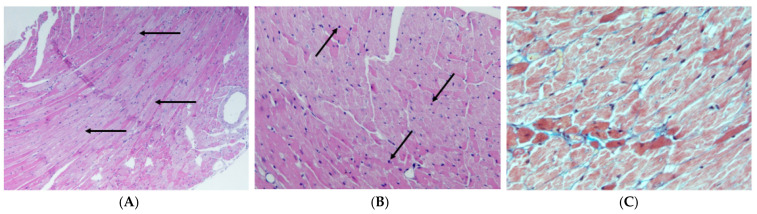
Histological sections of left ventricular myocardium from 14-month-old db/db mice (unpublished data by the authors). (**A**) Death of cardiomyocytes: areas of necrosis (arrows) appear lighter than living cells. Haematoxylin and eosin stain, 100× magnification. (**B**) Mononuclear leukocyte infiltration: leukocyte nuclei are indicated by arrows. Haematoxylin and eosin stain, 200× magnification. (**C**) Stromal fibrosis: Masson’s trichrome stain (collagen is stained blue), 400× magnification.

Echocardiographic and invasive haemodynamic data confirm the presence of HFpEF characteristic features in both murine strains. In db/db mice, the development of first diastolic (increased early to late mitral inflow (E/A) ratio and early mitral inflow to mitral annulus relaxation velocity (E/e′) ratio, prolonged isovolumic relaxation time (IVRT), decreased tissue Doppler e′/a′ ratio) [78,86,88,89,91,92,93,104] and subsequently systolic (reduced shortening fraction, EF, velocity of circumferential fibre shortening) LV dysfunction [81,86] is typical. ob/ob mice also exhibit LV diastolic dysfunction (decreased E/A ratio), but there are conflicting data regarding systolic dysfunction [81,96,100,101,102]. Many studies in db/db mice [78,81,84,86,88,89,92,93,103,104] demonstrate echocardiographic and histological evidence of LV hypertrophy accompanied by the development of myocardial fibrosis (Figure 3). However, no confirmations were found in other studies [87,93]. Additionally, in female mice, myocardial fibrosis is much more pronounced than in male mice [84]. Some studies have reported reduced capillary density in the myocardium [88,93], while others have reported normal or even increased density [87,92]. ob/ob mice also demonstrate the signs of LV hypertrophy (by echocardiography and post-mortem heart weighing) [81,95,98]. However, in contrast to db/db mice, histological data are scarce and ambiguous. For example, according to some sources, ob/ob mice have no or negligible myocardial fibrosis [95,96]; according to other research, myocardial fibrosis is still present [102].

In these murine strains, the presence of LV diastolic dysfunction is confirmed by an increase in plasma BNP level, a biological marker of haemodynamic stress [92,100]. Increased expression of natriuretic peptide estimated by mRNA level has been reported in the myocardium of ob/ob mice [96]. In db/db female mice, there is a significant relative increase in myocardial BNP mRNA content with age which is not confirmed in males [84]. In contrast, another study describes reduced myocardial expression of natriuretic peptide in db/db mice [105].

It should be noted that db/db mice demonstrate sex differences in the development of HFpEF symptoms. Thus, female mice have higher blood pressure levels and greater weight gain than males. Moreover, they have more pronounced LV remodelling processes (more pronounced LV hypertrophy and diastolic dysfunction) [88]. Such differences are indirectly in line with what is observed in humans where women are more likely to have severe HFpEF than men.

It is important that an increased cardiomyocyte residual stress induced by altered protein function increases LV myocardial stiffness. The studies report a decreased mRNA and protein content of SERCA2a in the db/db myocardium [78,83,106]. However, other studies confirm the molecular expression of SERCA2a in these mice that does not differ from that in healthy mice [92,93]. In ob/ob mice, the total amount of SERCA2a in the myocardium is not increased, although its decreased activity and increased oxidation are observed. In fact, this is associated with a decreased calcium ion transport from the cytoplasm to the sarcoplasmic reticulum and calcium ion accumulation in the cytoplasm [99] contributing to cardiomyocyte residual stress. A lot of studies [78,92,93] in db/db mice demonstrate that the total amount of the SERCA activity regulator protein phospholamban in the myocardium, as determined by western blotting, is comparable with the control ones, except for one study [106]. Phosphorylation of phospholamban removes its blocking effect on SERCA2a, favouring the fastest possible release of calcium ions into the sarcoplasmic reticulum and accelerating myocardial relaxation. Phosphorylation of phospholamban at serine 16 and threonine 17 has been revealed to be impaired in both db/db and ob/ob mice resulting in delayed calcium efflux, disturbed relaxation and increased LV myocardial stiffness [92,93,96]. In ob/ob mice, leptin administration restores the levels of phosphorylated phospholamban [98]. db/db murine cardiomyocytes are characterised by an impaired response to extracellular calcium and reduced contractility [85,92,106].

Changes in titin molecules which are intracellular spring elements responsible for myocardial stretching during diastole play an important role in increasing myocardial stiffness. In db/db mice, titin changes are mainly due to its hypophosphorylation rather than oxidation or isoform shift from highly extensible N2A molecules to more ‘rigid’ N2B molecules [89]. In both db/db and ob/ob mice, a change in MHC expression pattern is understood as an isoform shift from a faster α-isoform to a slower β-isoform [80,99]. Such alterations in myocardial MHC isoforms can be detected in 4-week-old mice [80,96]. However, the myocardial levels of MHC-β mRNA in db/db mice are higher in males, while females demonstrate a more pronounced relative increase with age [84]. Simultaneously, another study in db/db mice reports reduced mRNA content of MHC-α and MHC-β [105]. Additionally, in the myocardial tissue of ob/ob mice, the activity of protein kinase A which is responsible for the phosphorylation of titin and other cardiac proteins is reduced [98]. AMP-activated protein kinase levels are also reduced in the myocardium of db/db and ob/ob mice resulting in hypophosphorylation of titin and increased myocardial stiffness [78,79,87,91,93]. In contrast, no differences in the levels of the phosphorylated cardiac proteins myosin-binding protein C [cMYBP-C] and troponin I are observed in db/db mice [87,92].

Notably, mice of both strains have signs of dysfunction in the NO-cGMP-PKG signalling pathway that plays an essential role in normal myocardial function and has numerous beneficial effects such as accelerating relaxation, reducing cardiomyocyte stiffness, inhibiting pro-hypertrophic signalling, improving mitochondrial function, etc. [107]. Endothelium-dependent vasodilation is mediated by NO which is a potent relaxing factor. In the myocardium of db/db mice, both NO and nitrate levels (reflecting NO content) are reduced in comparison to those of healthy mice [91,92]. In db/db mice, there is a switch from NO-dependent to H_2_O_2_-dependent vasodilation contributing to impaired microvascular function [108]. The mRNA and protein content of different isoforms of NOS is altered in the myocardium of these mice, which is predominantly manifested by decreased expression of eNOS responsible for NO synthesis [79,105,108] and increased iNOS activity which is responsible for ROS generation [79,108]. Indeed, the decrease of cGMP protein level and cGMP-dependent PKG activity in the myocardium of db/db mice is also observed [89]. An indirect confirmation of the reduced NO bioavailability in db/db mice is confirmed by the disturbed myocardial perfusion observed in ex vivo and in vivo experiments [108].

The evidence of reduced NO bioavailability and impaired signalling through the intracellular NO-cGMP-PKG pathway is also confirmed in ob/ob mice. In particular, they have reduced levels of L-arginine which is a substrate for NOS, and increased levels of the NOS inhibitor asymmetric dimethylarginine [ADMA] in plasma [100]. In addition, ob/ob mice exhibit a reduced nitrate/nitrite ratio in heart homogenates which is a marker of ROS accumulation and reduced NO production [109]. However, other studies demonstrate an increased NO content in the myocardium [97]. A high level of iNOS and a low level of phosphorylated eNOS have been observed in the myocardium of ob/ob mice [97]. Unlike Guo W. et al. [97], these data are not confirmed when assessing eNOS mRNA levels by RT-qPCR. The same study illustrates a decrease in the content of neuronal NOS (nNOS) in the myocardium which can be restored after leptin administration [109].

Moreover, coronary microvascular dysfunction is observed in both ob/ob and db/db mice, most likely as a consequence of vascular tone-impaired regulation induced by reduced NO bioavailability [100]. To date, nitrosative/oxidative stress is a highly evident component of HFpEF pathophysiology [110] and its signs are found in the myocardium of both db/db and ob/ob strains. For example, an increase in NOX activity has been demonstrated in the myocardium of db/db mice. As a result, the binding of NO to superoxide (O_2_^−^) and subsequent formation of the cytotoxic reactive oxidant peroxynitrite (ONOO-) leads to protein dysfunction [78,103]. On top of that, levels of peroxynitrite are found to be elevated in the myocardium of both mouse strains [97,103]. The amount of protein carbonyls is increased in the myocardium of ob/ob mice indicating protein damage induced by oxidative stress [99]. High levels of the NOX1 and gp91phox (NOX2) subunits of NOX have been demonstrated in the myocardium of db/db mice [98]; however, mRNA content of the NOX2 subunit is higher in females [84]. Moreover, high expression of the membrane NOX subunits p47phox and gp91phox in ob/ob mice is revealed [99], although other studies have not confirmed this at either protein or mRNA level [109]. Nevertheless, increased activity of xanthine oxidoreductase [XOR] which is another likely source of ROS has been observed in ob/ob mice [109].

Furthermore, myocardial levels of the antioxidant glutathione (GSH), attributed to scavenging excess ROS, are reduced in db/db mice [103]. Glutathione is oxidised to glutathione disulfide (GSSG) during peroxide detoxification. A decrease in the GSH/GSSG ratio indicating high oxidative stress activity is also shown in the myocardium of ob/ob mice [99,109]. In addition, the expression of superoxide dismutase 1 and heme oxygenase 1 which provide protection against oxidative stress is increased in the myocardium of db/db mice [87]. In turn, the activity of catalase which has antioxidant properties, is increased in db/db myocardium [84].

It should be also noted that oxidative stress results in lipid peroxidation. Thus, the levels of malonic dialdehyde, the end product of lipid peroxidation, and non-esterified fatty acids which are markers of oxidative stress are increased in db/db mice [84]. Remarkably, the levels of these substances are higher in female mice indicating that females undergo more oxidative stress than males [84]. Moreover, the increased levels of malonic dialdehyde are also confirmed in the myocardium of ob/ob mice [99].

In accordance with the experiments results, in the myocardium of db/db mice signs of increased endoplasmic reticulum stress are observed in the form of increased expression of phosphorylated protein kinase RNA-like endoplasmic reticulum kinase [PERK], inositol-requiring protein-1α [IRE-1], and eukaryotic initiation factor 2 [eIF2α] [85], or the unfolded protein response in the form of increased protein concentration of GRP78/94, XBP-1, and C/EBP homologous protein [90]. Similarly, signs of endoplasmic reticulum stress and unfolded protein response, i.e., the presence of phosphorylated PERK, eIF2α, and C/EBP homologous protein, are also observed in ob/ob mice [97].

It is well-known that ROS are generated in the mitochondria. In keeping with the above findings, db/db mice demonstrate signs of mitochondrial dysfunction, mainly at the level of complex I, as evidenced by increased levels of H_2_O_2_ (produced by complex I) and superoxide in mitochondria. Thus, an increased permeability of the mitochondrial membrane contributes to the uncoupling of oxidative phosphorylation and ATP synthesis and leads to the accumulation of ROS in cardiomyocytes [91,103]. There is also evidence of reduced enzymatic activity of complex III of the electron transport chain (the main source of O_2_^−^) in these mice [103], although this has not been fully confirmed [91].

#### 2.1.2. HFD

Notably, C57BL/6J mice that are maintained on an HFD develop obesity [52,53,77,80,111,112,113,114,115,116,117,118,119,120,121,122,123] as glucose tolerance is impaired and insulin resistance results in elevated blood glucose and insulin levels [81,117,118,119,120,121,123,124]. These mice also have elevated blood levels of leptin which allows us to draw some parallels with obese humans [120,122]. Williams T.D. et al. highlight the development of arterial hypertension as one of the underlying conditions of this mouse model [116]. However, some authors have no confirmation of these data [124,125]. Such differences in blood pressure can be explained by the different HFD duration and the age of the mice. In all these studies, mice were switched to an HFD at the same age (5–6 weeks), but were followed for 12–15 weeks in one case [116] and for only 8 weeks in others [124,125]. It is possible that the increase in blood pressure in these mice occurs at a later age and with a longer period of time on a diet.

Yet, it is evident that HFD-fed mice show signs of systemic and local inflammation, the most important pathophysiological mechanism in the development and progression of HFpEF. In particular, they have an increased concentration of IL-6 in plasma [122] and NF-κB content in myocardial tissue [126] as well as increased IL-1β, IL-6, matrix metalloproteinase-9, plasminogen activator inhibitor-1, and adiponectin mRNA levels [120]. In fact, plasminogen activator inhibitor-1 is a key protein secreted by metabolically unhealthy visceral adipose tissue; its activation may contribute to HFpEF via accelerated ageing, inflammation, visceral adiposity, and impaired metabolism [127]. Moreover, histological examination demonstrates inflammatory cell infiltration in the myocardium and liver of these mice [117].

According to echocardiography, magnetic resonance imaging, and invasive haemodynamic studies, HFD mice demonstrate signs of LV diastolic dysfunction (decreased mitral velocity E, E/A and e′/a′ ratios, increased E/e′ ratio, prolonged IVRT) after 8 weeks on an HFD. At the same time, systolic function either is not altered significantly or worsened (reduced shortening fraction and EF) [81,119,121,123,124,126]. In addition to LV diastolic dysfunction, LV hypertrophy is identified according to echocardiography, magnetic resonance imaging, post-mortem weighing and histological examination in HFD-fed mice as well as myocardial fibrosis according to histological examination [119,121,123,124,125,126]. Within this concept, some researchers have demonstrated increased content of the pro-fibrotic factors TGF-β and phosphorylated Smad3 in the myocardium and decreased content of the anti-fibrotic factors bone morphogenetic protein-2 and phosphorylated Smad1/5 [120].

In HFD-fed mice, cardiac protein function has not been investigated as extensively as in db/db and ob/ob strains. Nevertheless, similar phenomena can be observed among the three models. For instance, the expression of SERCA2a is not significantly altered in all three models. However, the level of phosphorylated phospholamban which regulates SERCA2a and cardiac troponin T activity in the myocardium is reduced [121].

Furthermore, the signs of altered NO-cGMP-PKG signalling in the myocardium are proved in HFD-fed mice bringing this model closer to the db/db and ob/ob strains. Specifically, the content of nNOS and phosphorylated eNOS (with normal levels of total eNOS) is suppressed in HFD-fed mice [124,126]. Additionally, these mice have impaired endothelium-dependent vasodilation [122,128] and increased myocardial mRNA levels of the endothelial dysfunction markers endothelin-1 and angiotensin II type 1 receptor [120].

In addition to the db/db and ob/ob strains, HFD-fed mice also demonstrate signs of oxidative stress. For instance, they have increased levels of ROS and nitrotyrosine in the cytoplasm and mitochondria of cardiomyocytes, and high content of NOX1, NOX2 and NOX4 in the myocardium [120,122,124,126]. To compensate, myocardial antioxidants such as superoxide dismutase may be accumulated [121]. However, in other studies, superoxide dismutase levels remain unchanged [123,126] although another antioxidant, GSH, is decreased [123].

HFD-fed mice have pathological changes in the structure of cardiomyocyte mitochondria and an increased ratio of intracellular nicotinamide adenine dinucleotide phosphate [NADH] to nicotinamide adenine dinucleotide+ [NAD+] levels in the myocardium. This is indicative of mitochondrial oxidative stress and points to disturbances in the electron transport chain as a major source of ROS in the myocardium [124]. Other research in HFD-fed mice indicates that the permeability of myocardial mitochondrial membranes remains unchanged. The expression of uncoupling protein 3 [UCP3] crucial for electron transport chain disruption and oxidative phosphorylation uncoupling is similar to controls. Additionally, oxygen consumption in isolated cardiac mitochondria is not reduced [121]. Besides the myocardium, aortic protein levels of gp91phox and p47phox, subunits of NOX, are increased in these mice [122].

In obesity, HFD-fed mice exhibit a variety of metabolic disorders: plasma cholesterol, uric acid, triglycerides, and fatty acids; LDL levels are elevated compared to mice fed a healthy diet [117,118,119,121,122]. Myocardial lipid accumulation and fatty deposits in the liver (signs of steatosis) are also observed in this mouse strain [117,118,119,121]. Noticeably, activation of pro-apoptotic pathways is a molecular hallmark of HFD-fed mice. In particular, an increased expression of the pro-apoptotic *Bax* and caspase-3 and a decrease in the anti-apoptotic B-cell lymphoma 2 [*Bcl-2*] as well as an increase in pro-apoptotic procaspase-9 and caspase-9 expression are found in the myocardium of these mice [120,121].

### 2.2. Hypertension-Induced HFpEF Models

It has been established that arterial hypertension is a major risk factor for HFpEF, influencing both its development and prognosis.

#### 2.2.1. DOCA + High Salt Diet + Unilateral Nephrectomy [DOCA]

The DOCA model mimics the development of arterial hypertension as a result of mineralocorticoid excess reproducing only one component of HFpEF pathophysiology, specifically arterial hypertension. Given the simplicity, these mice can be employed to replicate multi-component models of HFpEF when combined with other risk factors.

The DOCA mice subjected to pellet implantation with a gradual release of DOCA and unilateral nephrectomy after being fed a high salt diet develop arterial hypertension within 3–7 days after surgery [129,130,131,132,133]. Simultaneously, it has been observed that male mice exhibit higher blood pressure levels compared to females [134]. Blood pressure elevation is associated with kidney damage [129] being more pronounced in male mice [134]. DOCA mice also demonstrate signs of pulmonary hypertension. This is most likely secondary to heart failure (left-sided pulmonary hypertension) [129]. No differences in body weight are observed in this mouse model [135]. Although initially these mice were used exclusively for hypertension modelling, they now are often considered as one of the relevant HFpEF models. This can be explained by the fact that the heart is a typical target organ of arterial hypertension, and hypertension itself is a major risk factor for the development of HFpEF [136].

Echocardiography and invasive haemodynamic studies show these mice have LV diastolic dysfunction (low e′ velocity, high E/e′ ratio and prolonged IVRT), sometimes with mild systolic dysfunction (low EF) [129,132,133,137]. Additionally, LV hypertrophy being more pronounced in male mice and myocardial fibrosis not being examined in detail in all studies are reported according to echocardiography, post-mortem weighing, and histological examination [129,130,131,132,134,138]. Moreover, one study reports higher TGF-β expression in male mice myocardium [134]. Some authors demonstrate lower myocardial capillary density [129], but others fail to confirm this [138].

Previously, the DOCA model considered only mechanical damage caused by volume overload of target organs including the heart. Similar to patients with arterial hypertension, these mice demonstrate signs of myocardial inflammation. For example, pro-inflammatory cytokines mRNA such as IL-1β, IL-6, IL-10, TNF, and MCP-1 (only in male mice) are detected alongside macrophage accumulation in the myocardium [129,130,134]. Furthermore, these mice exhibit signs of vascular inflammation as manifested by increased IL-1β, TNF, and MCP-1 mRNA content in the aortic wall [131].

In addition to the echocardiography and histological data, other signs of HFpEF have been established in this model, in particular, an increased expression of the haemodynamic stress marker atrial natriuretic peptide (ANP) in the myocardium [129,134,139]. Furthermore, male mice show a greater relative increase in ANP than females [134]. One study reported an increase in BNP expression [129], while other studies have not confirmed this [134,139].

Regarding myocardial protein dysfunction, the DOCA model has not been investigated in detail. However, one study has reported a down-regulation of phospholamban phosphorylation [133]. The signs of the NO-cGMP-PKG pathway dysfunction in the myocardium, i.e., decreased NO levels [140] and increased levels of oxidised tetrahydrobiopterin (a cofactor of eNOS) are confirmed in DOCA mice. As such, these alterations promote subsequent substrate uncoupling of eNOS [133] and expression of iNOS [132], and reduced expression of eNOS [140] (although the latter has not been demonstrated in all studies) [132]. Moreover, a low level of eNOS mRNA has also been found in the aorta [131].

As anticipated, DOCA mice demonstrate signs of oxidative stress in the myocardium and vessels. In particular, the levels of superoxide and nitrotyrosine are increased in their myocardium [132,133,140]. However, myocardial mRNA levels of p22phox, gp91phox (NOX2) and NOX4 are not significantly altered distinguishing these mice from the above-described models [138]. Some authors suggest that NOS may be the main source of superoxide in DOCA mice [133]. Thus, in iNOS knockout mice, myocardial relaxation is less impaired, myocardial nitrotyrosine content is lower, and eNOS expression tends to be higher compared to non-knockout mice. Therefore, these findings indirectly support the involvement of iNOS in ROS production [132]. In addition, superoxide levels and the expression of gp91phox and p22phox in the aorta are also increased in this murine model [131,141,142]. Some authors propose that these changes are more pronounced in male mice than in female mice which were similar to the control group in several parameters [134]. This is not exactly in line with what is observed in HFpEF patients where women with the cardiometabolic phenotype are more likely to have a higher incidence and severity of HFpEF compared to men.

This model may better suit the cardiorenal phenotype of HFpEF, characterised by high blood pressure and kidney damage which occurs equally in men and women. However, it is too early to draw any conclusions about the suitability of this model for HFpEF as it has not been well studied as a model for HFpEF. Furthermore, it should be noted that the C57BL/6 mouse strain is quite resistant to the development of arterial hypertension and kidney damage [135]. In this case, the 129/Sv murine strain is most susceptible, with arterial hypertension, LV hypertrophy, and kidney damage being more pronounced than in the C57BL/6 strain [135].

#### 2.2.2. Angiotensin II (Angiotensin II Infusion) [Ang II]

Ang II mice are implanted with an osmotic mini-pump that infuses angiotensin II. The development of arterial hypertension occurs within one week of starting angiotensin II infusion [142,143]. Ang II mice are also not well characterised as a model of arterial hypertension as angiotensin II infusion alone may not be sufficient to mimic arterial hypertension because of the resistance of the C57BL/6 strain to hypertensive organ damage. However, higher levels of blood pressure are observed in the Ang II model compared to the DOCA model as pointed out by some authors [139].

The signs of LV diastolic dysfunction (verified by IVRT prolongation) without systolic dysfunction are described in the Ang II model even with the administration of low doses of angiotensin II insufficient to raise blood pressure [144]. This observation suggests that inflammation and/or metabolic disturbances, rather than mechanical or haemodynamic factors (increase in afterload and preload) are crucial in developing HFpEF. In the above experiment [144], LV filling abnormalities at low doses of angiotensin II infusion may be due to its pro-inflammatory properties.

Consistent with the prior studies, angiotensin II also stimulates NOX in inflammatory cells leading to ROS accumulation. Adoptive transfer of T-cells lacking the angiotensin type I receptor blunts angiotensin II-dependent hypertension and reduces aortic superoxide production [142]. Myocardial infiltration by CD68+ macrophages and increased myocardial protein expression of the pro-inflammatory cytokines TNF and IL-1β is also observed in the Ang II model [143]. This provides further evidence for the involvement of inflammation in the development of HFpEF.

As in other models of HFpEF, histological examination reveals hypertrophy and fibrosis of the LV myocardium [143,144]. Furthermore, fibrosis is more pronounced in the Ang II model than in the DOCA model [140]. In addition, the expression of ANP and BNP and the MHC-α/MHC-β ratio are increased in the myocardium to a greater extent than in the DOCA mice [139]. These mice also show impaired endothelium-dependent vasodilation and increased ROS production in the aorta [142,143].

#### 2.2.3. DOCA + High Salt Diet + Angiotensin II + Unilateral Nephrectomy [DOCA + Ang II]

The DOCA + Ang II model has been developed in order to overcome the resistance of the C57BL/6 strain to hypertensive organ damage, in particular, kidneys. It has also been understudied and poorly characterised as a model of HFpEF, as are all hypertensive models. Obviously, many abnormalities appear to be more pronounced in the DOCA + Ang II model compared to the DOCA or Ang II models alone [139]. They include high blood pressure, histochemical and laboratory signs of renal damage, severity of LV hypertrophy and fibrosis, increase in myocardial expression of ANP and BNP, and MHC-β/MHC-α ratio [139].

#### 2.2.4. Transverse Aortic Constriction (TAC)

TAC mice undergo ligation of the aortic arch between the bronchopulmonary trunk and the left common carotid artery to induce the development of pressure overload-induced cardiac hypertrophy and HF. The body weight of TAC mice is comparable to that of intact animals [145]. Yet, the TAC model is widely utilised to study arterial hypertension. Moreover, it is actively used as a model for HFpEF demonstrating LV filling abnormalities. The obvious disadvantage of the TAC model as well as other above-mentioned models of HF induced by arterial hypertension is the reproduction of only one clinical aspect of HFpEF, specifically pressure overload. In some hypertensive models, TAC mice show signs of pulmonary oedema, fibrosis, and pulmonary vasculature remodelling (according to the histological study) [146]. These changes are likely to result from LV dysfunction.

Data published indicate that the TAC model typically develops LV diastolic dysfunction shown by a shortened deceleration time of early diastolic mitral flow followed by systolic dysfunction indicated by reduced EF and shortening fraction [132,140,141,142,143,144,145]. It should be noted that most of these studies did not evaluate the most important diastolic parameters such as the E/A, E/e′, e′/a′ ratios, and IVRT. Moreover, LV hypertrophy is confirmed in TAC mice according to echocardiography, post-mortem weighing and histological examination [138,145,146,147,148,149,150,151].

Histological studies also conclude the development of myocardial fibrosis in TAC mice providing an increased expression of TGF-β by myocardial cells [138,145,147,149]. In addition, there is a decrease in the density of capillaries in the myocardium [138]. Increased production of ANP and BNP in the myocardium is another sign of HF [147,149].

Limited data exist on contractile protein expression patterns for the TAC model. In particular, an increase in MHC-β content is found indicating a shift from fast MHC-α to slower MHC-β isoform. It should be noted that these changes have also been observed in other HF models as well [147]. In addition, phosphorylation of ryanodine receptor type 2 [RYR2], the major intracellular calcium release channel in the cardiac sarcoplasmic reticulum, and the calcium transport regulatory protein phospholamban are reduced [151].

The TAC model also demonstrates some evidence of NO-cGMP-PKG pathway dysfunction in the myocardium: although total eNOS and nNOS levels are comparable with the control ones, the phosphorylation of eNOS on Ser114 and nNOS on Ser1412 is reduced [145].

Additionally, this model has evidence of myocardial oxidative stress: increased ROS, decreased nitrate/nitrite ratio (a marker of increased ROS and decreased NO production), activity of the antioxidant enzyme superoxide dismutase, and GSH/GSSG ratio [145,147]. Thus, this evidence suggests a depletion of antioxidant systems [149].

#### 2.2.5. TAC + DOCA

TAC + DOCA mice undergo ligation of the aortic arch followed by the implantation of a DOCA pellet. The development of diastolic dysfunction and increased LV filling pressure, but preserved systolic function is characteristic of this model [152,153,154]. Obviously, such changes are more pronounced in TAC + DOCA than in TAC or DOCA models alone [138].

Furthermore, histological studies of the myocardium reveal hypertrophy, fibrosis, and reduced capillary density [138,153,154]. These phenomena occur in patients with HFpEF [155].

It is important to note that TAC + DOCA mice gather reduced exercise tolerance over time [152,154]. In addition, this model also shows signs of oxidative stress in the myocardium manifested by an increase in mRNA levels of p22phox, gp91phox (NOX2) and NOX4 (NOX subunit) [138].

### 2.3. HFpEF as a Result of Accelerated Ageing (The Senescence-Accelerated Mice)

Ageing is a significant risk factor for heart failure, with HFpEF becoming more common due to age-related conditions.

The SAMP8 murine strain derived from AKR/J mice is known for accelerated senescence, with an average lifespan of 9.7 months [156]. There are over a dozen accelerated ageing lines, but SAMP8 is commonly used. Six-month-old SAMP8 mice exhibit early indicators of ageing [157]. Unlike humans, where age correlates with arterial hypertension and HFpEF risk, blood pressure is not elevated [157]. Therefore, it was initially utilised only to study the ageing process.

Despite the fact that ageing affects the heart, being an independent risk factor for the development of HFpEF, limited information regarding HF pathophysiology is provided in this model. In particular, there are no data on whether myocardial proteins involved in contraction/relaxation and regulation of intracellular calcium turnover are altered.

Following echocardiography and invasive haemodynamic study, LV diastolic dysfunction (decreased E/A and e′/a′ ratios) is developed by 6 months of age in SAMP8 mice, but the systolic function remains preserved [157].

According to post-mortem weight data, LV hypertrophy is proved in this strain [157,158]. Histological examination reveals myocardial fibrosis [157,159], and up-regulation of pro-fibrotic TGF-β is confirmed in heart tissue [157]. In addition, the ANP mRNA content is increased in the myocardium of SAMP8 mice [158].

As stated before, ageing is associated with the development of chronic inflammation. In the SAMP8 mice, signs of inflammation can be detected in the myocardium, in particular, increased mRNA levels of the pro-inflammatory cytokines TNF, IL-1 and IL-6 and increased mRNA and protein levels of the p50 and p52 subunits of NF-κB [159,160]. In contrast, other data have shown that these mice are comparable with control ones in terms of NF-κB activity and expression of TNF and MCP-1 [158].

The SAMP8 strain also demonstrates signs of myocardial NO-cGMP-PKG pathway dysfunction (down-regulation of eNOS, and up-regulation of iNOS) as well as evidence of oxidative damage to cardiomyocyte DNA [159,160]. On the other hand, there is evidence for an elevated expression of myocardial haem oxygenases 1 and 2 which may protect cells from the pathological action of ROS [160].

Senescence-accelerated mice often develop both senescent amyloidosis and secondary AA amyloidosis [161]. They are susceptible to lymphoma, inflammation (colitis, pneumonia, abscesses), and renal failure [161]. The molecular features of the SAMP8 model are a decrease in the anti-apoptotic B-cell lymphoma 2 [*Bcl2*] expression and an increase in the pro-apoptotic Bcl-2 associated X-protein [*BAX*] expression [161] as well as an increase in the activity of the pro-apoptotic caspase 3 [159,160,161].

Table 1 summarises HFpEF manifestations in mouse models.

## 3. Limitations of HFpEF Murine Models

According to data published, the db/db and ob/ob lines most accurately replicate the cardiometabolic characteristics typical of HFpEF among all the murine models. These strains represent the most well-characterised models examined in this review. Consequently, they illustrate sex differences in the onset and progression of HFpEF that closely resemble its clinical course in humans. The db/db strain shows a rapid development of pathological changes. In contrast, people with HFpEF experience a slower progression of these changes. ob/ob mice show slower pathological changes making them more comparable to humans. Another drawback of the db/db and ob/ob strains is their infertility which creates challenges in breeding these models. In the ob/ob strain, this issue can be addressed with leptin administration. The HFD model is simpler and more accessible resembling natural conditions, but it has been studied less extensively than the db/db and ob/ob strains. Furthermore, HFD mice likely do not exhibit sex differences in pathological manifestations and thus, they may not accurately replicate the human clinical progression of HFpEF. Next, DOCA and Ang II models serve as representations of isolated arterial hypertension. Available data indicate that these models primarily involve vascular and renal systems rather than cardiac involvement; however, there are instances where inflammation is observed in the myocardium. The DOCA and Ang II models can be effectively utilised to investigate isolated arterial hypertension as a contributing factor to HFpEF. Further studies are needed to better characterise them. Aortic ligation models are the least characterised murine models for inflammation in HFpEF development. Thus, isolated aortic ligation is seen as a ‘mono’ model as it is neither ‘natural’ nor easy to perform. Although the addition of DOCA infusion to aortic ligation may have potential benefits, current data on the TAC + DOCA model are insufficient to assess its appropriateness for studying HFpEF. Finally, accelerated ageing models can study HFpEF’s age-related characteristics, but they remain poorly understood. Additionally, ‘genetically accelerated’ ageing is an artificial process, and these mice do not have T2DM, obesity, or arterial hypertension which are characteristics of patients with HFpEF. It is also noticeable that elderly patients with HFpEF and no cardiometabolic comorbidities are rarely seen in clinical practice.

Table 2 provides a summary of the reasons for the limited use of murine models in the study of HFpEF.

In summary, murine models of HFpEF do not fully reflect the cardiometabolic phenotype of HFpEF in humans, as evidenced by discrepancies between clinical trials and experimental data. Currently, research is being conducted on the use of SGLT2 inhibitors and GLP-1 receptor agonists in the treatment of HFpEF. Randomised clinical trials have shown SGLT2 inhibitors improve cardiovascular outcomes in HFpEF patients [162,163], although experimental results have been inconsistent.

In HFD mice, SGLT2 inhibitors effectively reduce diastolic dysfunction, myocardial hypertrophy and fibrosis, mRNA for inflammatory and fibrosis markers as well as myocardial ROS production [164,165]. In db/db and ob/ob mice, empagliflozin improves LV diastolic function and reduces oxidative stress, mitochondrial dysfunction and ANP and BNP mRNA, although its effect on myocardial hypertrophy and fibrosis is unclear [91,166,167]. However, in TAC mice, empagliflozin shows no impact on LV diastolic dysfunction or myocardial hypertrophy and fibrosis severity [168].

Recent clinical trials show GLP-1 receptor agonists are effective for obesity-related HFpEF [169]. In mice, these drugs are most effective with Ang II and HFD models. Liraglutide partially prevented LV diastolic dysfunction in AngII mice and improved it in HFD mice, also reducing cardiomyocyte hypertrophy and cardiac fibrosis, and enhancing exercise tolerance [170,171]. In accordance with the functional improvements, semaglutide treatment in aged female C57BL6/J mice with HFD and angiotensin II infusion resulted in reduced LV hypertrophy and fibrosis, decreased RNA levels of fibrosis markers, and lower levels of inflammatory markers in the myocardium [172]. However, effects were not reproducible in the db/db model [171].

The presence of this class of drugs in HFD mice, as opposed to their absence in models of genetic obesity, indicates pathophysiological differences in the development of HFpEF between mice and humans. This distinction impacts the translational potential of experimental (mouse) data.

## 4. Conclusions and Future Perspectives

**Figure 4:**
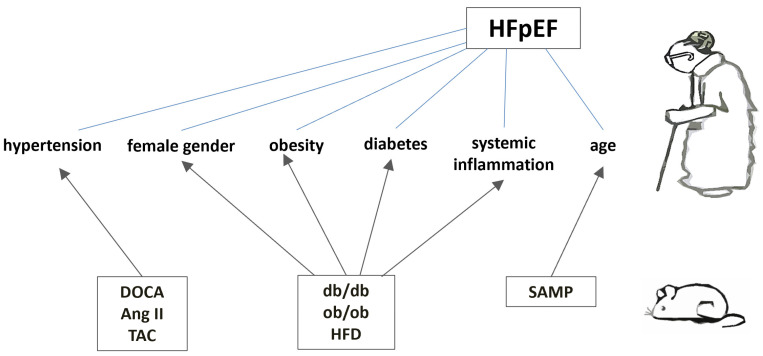
Pathophysiological Mechanisms in Cardiometabolic HFpEF and related murine models.

In conclusion, HFpEF remains a complex and multifaceted condition with significant clinical and social impact. A better understanding of the mechanisms underlying the pathophysiology of HFpEF depends on the quality and comprehensiveness of basic, translational, and clinical research. The murine models described in this review reproduce classic risk factors for the development of HFpEF, such as arterial hypertension, obesity, T2DM and ageing, and will undoubtedly contribute to a better understanding of different aspects of HFpEF and help develop new treatment strategies (Figure 4). However, these models have several disadvantages. First, they do not replicate additional cardiac and non-cardiac impairments found in human HFpEF, such as atrial fibrillation, renal dysfunction, and chronic obstructive pulmonary disease [57]. Second, the lack of extensive characterisation of certain models hinders their potential for further investigation as models for HFpEF. Additionally, several murine models may develop LV systolic dysfunction over time which is more typical of ‘mono’ models with isolated pressure overload or insulin resistance. It is worth noting that in complex models, LV systolic function typically remains preserved into advanced age [59].

The figure illustrates the key pathophysiological mechanisms involved in the cardiometabolic phenotype of heart failure with preserved ejection fraction (HFpEF). Understanding these mechanisms is crucial for developing targeted therapeutic strategies for HFpEF. Abbreviations are the same as those in Table 1.

Further research should explore these models and their translational potential to bridge the gap between preclinical findings and clinical applications.

## Figures and Tables

**Table 1:** HFpEF manifestations in mouse models.

	db/db	ob/ob	HFD	DOCA	Ang II	DOCA + Ang II	TAC	TAC + DOCA	SAMP
Systemic inflammation signs	+			no data					
Myocardial inflammation signs	+				+/− (limited data)	no data			+
Myocardial hypertrophy	Described for all models								
Myocardial oxidative stress	+				no data		+/− (limited data)		
Myocardial NO-cGMP-PKG signalling pathway dysfunction	+	+/− (limited data)			no data		+/− (limited data)	no data	+/− (limited data)
Structural and functional alterations in cardiomyocyte contractile proteins	+		+/− (limited data)					no data	
Myocardial fibrosis signs	+	+/− (limited data)	+						
Diastolic dysfunction signs (according to echocardiographic data)	+				+/− (limited data)	no data	+/− (limited data)		
Elevated ANP/BNP in myocardial tissue and/or blood	+		no data	+	+/− (limited data)		+	no data	+/− (limited data)
Gender differences	+ (females show more pronounced changes)	+/− (limited data, females show more pronounced changes)	no data	+/− (limited data, males show more pronounced changes)	no data				

ANP—atrial natriuretic peptide; BNP—brain natriuretic peptide. Murine models: Ang II—angiotensin II infusion; db/db—leptin receptor-deficient strain; DOCA—chronic subjection to deoxycorticosterone acetate; HFD—high-fat diet; ob/ob—leptin-deficient strain; SAMP—senescence-accelerated prone strain; TAC—transverse aortic constriction.

**Table 2:** Limitations of HFpEF murine models.

	Model Limitations in Data Translation to Humans	Challenges of Practical Application
db/db, ob/ob	Rather rapid development of obesity and insulin resistance, which is unlike typical human conditions	Homozygote infertility increases breeding costs
HFD	Possible development of systolic dysfunction	Duration of follow-up
DOCA, Ang II, DOCA + Ang II	Elevated blood pressure is a significant factor in the onset of human damage, including the progression to heart failure	Duration of follow-up (the C57BL/6 strain is fairly resistant to arterial hypertension development and hypertensive damage)
TAC	Primarily hypertensive organ damage, with indication of systolic dysfunction	The need for surgical intervention
TAC + DOCA	Primarily hypertensive organ damage	
SAMP	Early manifestations of inflammatory damage in various organs, neurodegeneration, age-associated lymphoproliferative conditions preceding the development of heart failure	Difficulties in breeding and housing animals due to short fertility period and high mortality rate

Abbreviations are the same as those in Table 1.

## Abbreviations

The following abbreviations are used in this manuscript:

ADMA	Asymmetric dimethylarginine
ANP	Atrial natriuretic peptide
Bcl-2	anti-apoptotic B-cell lymphoma 2
BNP	Brain natriuretic peptide
cGMP	Cyclic guanosine monophosphate
cMYBP-C	Myosin-binding protein C
DOCA	Deoxycorticosterone acetate
eIF2α	Eukaryotic initiation factor 2
eNOS	Endothelial nitric oxide synthase
GSH	Glutathione
GSSG	Glutathione disulfide
HF	Heart failure
HFD	High-fat diet
HFpEF	Heart failure with preserved ejection fraction
HFrEF	Heart failure with reduced ejection fraction
IL	Interleukin
IRE-1	Inositol-requiring protein-1α
iNOS	Inducible nitric oxide synthase
IRS-1	Insulin receptor substrate-1
IVRT	Isovolumic relaxation time
LV	Left ventricular
MCP-1	Monocyte chemotactic protein-1
MHC	Myosin heavy chain
NAD+	Nicotinamide adenine dinucleotide phosphate
NADH	Nicotinamide adenine dinucleotide phosphate
nNOS	Neuronal nitric oxide synthase
NO	Nitric oxide
NOX	Nicotinamide adenine dinucleotide phosphate oxidase
NT-proBNP	N-terminal fragment of brain natriuretic hormone precursor
PERK	Protein kinase RNA-like endoplasmic reticulum kinase
PKG	Protein kinase G
ROS	Reactive oxygen species
SAMP	Senescence-accelerated prone strain
SERCA	Sarco-endoplasmic reticulum Ca^2+^-ATPase
SIRT	Silent mating type information regulation 2 homolog 1
T2DM	Type 2 diabetes mellitus
TAC	Transverse aortic constriction
TGF	Transforming growth factor
TNF	Tumour necrosis factor
XOR	Xanthine oxidoreductase
